# Structural Insight Into Conformational Changes Induced by ATP Binding in a Type III Secretion-Associated ATPase From *Shigella flexneri*

**DOI:** 10.3389/fmicb.2018.01468

**Published:** 2018-07-02

**Authors:** Xiaopan Gao, Zhixia Mu, Xia Yu, Bo Qin, Justyna Wojdyla, Meitian Wang, Sheng Cui

**Affiliations:** ^1^MOH Key Laboratory of Systems Biology of Pathogens, Institute of Pathogen Biology, Chinese Academy of Medical Sciences & Peking Union Medical College, Beijing, China; ^2^Swiss Light Source, Paul Scherrer Institute, Villigen, Switzerland

**Keywords:** *Shigella flexneri*, T3SS, type III secretion-associated ATPase, F/V-type ATPase, proton motive force (PMF), ATP analog

## Abstract

Gram-negative bacteria utilize the type III secretion system (T3SS) to inject effector proteins into the host cell cytoplasm, where they subvert cellular functions and assist pathogen invasion. The conserved type III-associated ATPase is critical for the separation of chaperones from effector proteins, the unfolding of effector proteins and translocating them through the narrow channel of the secretion apparatus. However, how ATP hydrolysis is coupled to the mechanical work of the enzyme remains elusive. Herein, we present a complete description of nucleoside triphosphate binding by surface presentation antigens 47 (Spa47) from *Shigella flexneri*, based on crystal structures containing ATPγS, a catalytic magnesium ion and an ordered water molecule. Combining the crystal structures of Spa47-ATPγS and unliganded Spa47, we propose conformational changes in Spa47 associated with ATP binding, the binding of ATP induces a conformational change of a highly conserved luminal loop, facilitating ATP hydrolysis by the Spa47 ATPase. Additionally, we identified a specific hydrogen bond critical for ATP recognition and demonstrated that, while ATPγS is an ideal analog for probing ATP binding, AMPPNP is a poor ATP mimic. Our findings provide structural insight pertinent for inhibitor design.

## Introduction

Shigellosis caused by *shigella* species is a leading cause of diarrheal disease worldwide and the second leading cause of death in children aged 1–4 years living in low-income and middle-income countries, there are roughly 1,64,000 annual deaths attributable to shigellosis (Kotloff et al., 2018). *Shigella* species are facultative intracellular pathogens that cause diarrhea by invading human host cells, evade host immune responses (Killackey et al., 2016). Like many Gram-negative bacterial pathogens, a key component of *Shigella* pathogenicity depends on the presence of a type three secretion system (T3SS) (Schroeder and Hilbi, 2008; Puhar and Sansonetti, 2014).

The T3SS is a specialized apparatus employed by many Gram-negative bacteria and symbionts to inject effector proteins into host cells (Blaylock and Schneewind, 2005; Cornelis, 2006; Deng et al., 2017). The T3SS is crucial to the virulence of pathogenic bacteria in humans, animals and plants (Galan and Wolf-Watz, 2006; Cornelis, 2010). T3SSs are evolutionarily related to flagella and many substructures and components that are involved in assembly are highly conserved, which is built from more than 20 unique components assembled into a 3.5 megadalton syringe-like complex, including a cytosolic ATPase complex, a cytoplasmic ring (C-ring), an inner membrane export apparatus, a basal body, and a translocation pore that is in the host cell membrane (Kubori et al., 1998; Galán et al., 2014; Deng et al., 2017; Galán and Waksman, 2018). Upon delivery into the cytoplasm, effector proteins engage in various activities that subvert cellular functions and promote pathogen invasion.

The T3SS injectisome is able to recognize a wide variety of effector proteins, but its components are highly conserved among Gram-negative bacteria (Galán et al., 2014). The mechanism of effector recognition has not been fully elucidated. It is generally believed that effector proteins require customized guidance to enter the T3SS apparatus. This may be achieved either through a signaling peptide located at the N-terminus of the effector protein (Cornelis, 2003), or by a chaperone that specifically binds to the N-terminal region of the effector (Akeda and Galan, 2005; Birket et al., 2007; Lokareddy et al., 2010). The effector protein is then fitted into the central channel of the syringe-like complex that has a diameter of only ~20 Å, which is too narrow for most effector proteins. Therefore, the effectors must be unfolded before translocation (Akeda and Galan, 2005; Yip and Strynadka, 2006; Radics et al., 2014). Finally, the unfolded effector protein is passed through the channel connecting the bacterial cytoplasm and host cell cytoplasm, across the bacterial outer membrane and through the host inner membranes, covering a distance of ~600–800 Å. A highly conserved T3SS-associated ATPase InvC from *Salmonella* recognizes T3SS effector/chaperone complexes, strips the chaperones off secreted proteins and unfolds the effector proteins in an ATP-dependent manner, thereby preparing the substrates for secretion through the T3SS channel (Akeda and Galan, 2005), although it is dispensable for T3SS if proton motive force(PMF)across the cytoplasmic membrane is high enough (Erhardt et al., 2014). Therefore, the precise mechanism by which T3SS-associated ATPases hydrolysis supports secretion is unresolved. An appealing model is that the T3SS-associated ATPases are similar to ATP-driven translocases involved in substrate recognition, unfolding, and translocation of effector proteins (Akeda and Galan, 2005; Kato et al., 2015). The ATP-driven translocase belongs to the AAA+ superfamily, members of which assemble into a hexameric ring structure with a central pore. The ATP-driven translocase hydrolyses ATP to power conformational changes of the hexameric ring so that the protein substrate is unfolded and translocated through the pore of the ring (Sauer and Baker, 2011). Therefore, it was proposed that T3SS-associated ATPases also assemble into a homo-hexameric ring with a central pore through which substrates are funneled during the unfolding process, coupled with ATP hydrolysis that provides energy for pushing substrates through the secretion channel, thereby driving protein export (Akeda and Galan, 2005; Galán, 2008; Kato et al., 2015; Lee and Rietsch, 2015).

Recently, results from electron microscopy (EM), high-resolution cryo-electron tomography (cryo-ET) and biochemical studies demonstrated that the T3SS ATPase assembles into a homo-hexameric ring [(secretion and cellular translocation) SctN_6_/FliI_6_], and SctO/FliJ occurs in the central pore located on the cytoplasmic side of the T3SS apparatus, similar to the F/V-type ATPases (Galan and Wolf-Watz, 2006; Müller et al., 2006; Kazetani et al., 2009; Lorenzini et al., 2010; Ibuki et al., 2011; Kawamoto et al., 2013; Kishikawa et al., 2013; Radics et al., 2014; Hu et al., 2015, 2017; Imada et al., 2016). The extensive structural similarity between the T3SS SctN_6_SctO/FliI_6_FliJ (a Spa47_6_Spa13 homolog) ring complex and F/V-type ATPases suggests a similar mechanisms and a close evolutionary relationship (Minamino et al., 2008; Ibuki et al., 2011; Kawamoto et al., 2013; Minamino, 2014). The F/V-type ATPases couple ATP synthesis and hydrolysis to proton translocation across the membrane via a rotational catalysis mechanism. Stepwise rotation of the γ subunit in the middle of the ring is coupled with conformational changes of the β subunits and concomitant sequential ATP binding and hydrolysis (Noji et al., 1997; Yoshida et al., 2001). Ten years ago, Imada and colleagues suggested that conformational changes of the FliI ATPase are coupled with the ATP hydrolysis cycle (Imada et al., 2007). More recently, Morimoto and colleagues showed that ATP hydrolysis by FliI and subsequent protein export through the export gate are both coupled with inward-directed proton translocation through the T3SS gate (Morimoto et al., 2016). Since the T3SS cytoplasmic ATPase complex is structurally and functionally very similar to the extramembrane part of F/V-type ATPases (Imada et al., 2016). Therefore, the T3SS export apparatus presumably acts as an H^+^/protein antiporter to couple ATP hydrolysis with an inward-directed H^+^ flow through the gate with an outward-directed T3SS protein export (Morimoto et al., 2016). Although many structures of T3SS ATPases have been determined (Imada et al., 2007, 2016; Zarivach et al., 2007; Walker, 2013; Allison et al., 2014; Burgess et al., 2016a), none managed to capture the predicted hexameric ring structure at high resolution. Moreover, these structures failed to provide atomic details for ATP recognition- and ATP binding-induced conformational changes. It therefore remains unclear exactly how ATP binding and hydrolysis are coupled to support secretion or protein export.

Using a crystallographic approach, in the present study we reveal atomic details for ATP binding by the highly conserved T3SS ATPase Spa47 from *Shigella flexneri*. We captured snapshots that facilitate a complete description of nucleoside triphosphate recognition, including a bound slowly hydrolysable ATP analog ATPγS, a catalytically important Mg^2+^ ion and an ordered water molecule. Crystal structures of Spa47-ATPγS and unliganded Spa47 demonstrate that conformational changes in Spa47 are associated with ATP binding. Additionally, binding of ATPγS initiates a chain of movements that spreads from the ATP-binding pocket to the conserved luminal loop facing the predicted pore of the Spa47 hexamer. Our findings support the previously proposed mechanism of type III-associated ATPase function in protein export, and suggest that nucleotide-driven conformational changes are likely linked to the rotation of Spa13 similar to F/V-type ATPases, based on the fact that Spa13 is postulated to reside at the center of the Spa47 ATPase ring in a similar way to FliJ and InvJ (Ibuki et al., 2011; Cherradi et al., 2014; Hu et al., 2015, 2017). These results further imply that T3SS ATPases and F/V-type ATPases share a common evolutionary origin and exhibit similar mechanistic features.

## Materials and methods

### Cloning, protein expression, and purification

The cDNA encoding Spa47Δ1-83 (84–430 aa) was amplified from the *S. flexneri* pCP301 virulence plasmid using the polymerase chain reaction (PCR). The PCR product was digested by NdeI and XhoI enzymes, and the resulting DNA fragment was inserted into a pET28a vector for expression of the N-terminal 6 × His tagged protein. The full-length Spa47 was cloned into expression plasmid pTYB21 as described previously (Burgess et al., 2016b). Plasmids expressing full-length Spa47 mutants were prepared using site-directed mutagenesis (KOD Plus) following the manufacturer's instructions. The sequences of the mutants were confirmed using DNA sequencing. The plasmids encoding Spa47Δ1-83 and full-length Spa47 or its mutants were transformed into *E. coli* Rosetta™ strain (DE3) competent cells (Novagen) for expression. The bacterial cultures were grown in LB medium at 37°C. Induction was initiated by the addition of IPTG (0.5 mM for Spa47Δ1-83 and 1 mM for full-length Spa47) when the culture reached OD_600_ = 1.2. The bacterial culture was continued with shaking at 18°C overnight after induction. The bacterial cells were then harvested by centrifugation (5,000 rpm, 30 min) and stored at −20°C. To purify Spa47Δ1-83, the cell pellets were re-suspended in lysis buffer containing 20 mM Tris-HCl pH 8.0, 150 mM NaCl, 10 mM imidazole, and 4 mM β-mercaptoethanol and disrupted by ultrasonication on ice. The cell debris was removed by centrifugation at 13,000 rpm for 30 min. The crude lysate was loaded onto Ni-NTA resin (Invitrogen) pre-equilibrated with lysis buffer and eluted with 200 mM imidazole. The 6 × His tag was finally cleaved by thrombin digestion in dialysis buffer (20 mM Tris-HCl pH 8.0, 75 mM NaCl). The non-tagged Spa47Δ1-83 was loaded into a Hitrap Q HP column (GE Healthcare) and eluted with a linear gradient of 75–1,000 mM NaCl. The final step of purification was size exclusion chromatography using a Superdex 75 HR 10/30 column (GE Healthcare) equilibrated with 20 mM Tris-HCl pH 8.0, 100 mM NaCl, and 2 mM DTT. The selenomethionine-substituted Spa47Δ1-83 was prepared by expressing the protein in the B834 (DE3) strain grown in LeMASTER medium containing L-selenomethionine. The protein was purified using the same protocols as the native protein. The purification of full-length Spa47 and each of the Spa47 mutants were performed as previously described (Burgess et al., 2016b)

### Crystallization and structure determination

Crystallization trials of Spa47Δ1-83 were conducted in a hanging-drop vapor-diffusion system at 22°C. The protein was concentrated to approximately 10 mg/ml and 5–10 mM MgCl_2_ was added before the experiments. The optimized conditions for crystallization were achieved by mixing 1 μl of buffer containing 0.1 M bicine, pH = 8.0, 1.4 M ammonium sulfate and 1 μl of protein solution. Single crystals of Spa47Δ1-83 reached an average size of 0.3–0.5 mm after 48 h of incubation. Crystal-soaking experiments were conducted by transferring Spa47Δ1-83 crystals to a drop of reservoir buffer containing 5 mM ATP analog (ATPγS, AMPPNP) and incubating for 8 h at 22°C. The cryocooling of the crystals was performed by soaking the crystals in reservoir buffer containing 10% ethylene glycol 30–60 s before flash freezing in liquid nitrogen.

X-ray diffraction experiments were conducted at beamline BL17U at the Shanghai synchrotron radiation facility (SSRF), Shanghai, China and at SLS beamline X06DA at the Swiss light source, Paul Scherrer Institut, Villigen, Switzerland. Complete datasets for Spa47Δ1-83 crystals were collected using X-rays with a wavelength of 0.979 Å. The images were integrated and scaled using XDS (Kabsch, 2010). The AUTOSHARP/SHARP program was used to locate the Se atoms and calculate the initial phase to produce an interpretable electron density map (Vonrhein et al., 2007). The atomic model was built manually using the Coot program (Emsley et al., 2010) and refined using PHENIX (Echols et al., 2012). The final model had excellent refinement statistics and stereochemical quality. The structures of Spa47Δ1-83 in complex with ATP analogs, AMPPNP or ATPγS were solved by molecular replacement (McCoy, 2007) using the structures of unliganded Spa47Δ1-83 as the searching models. All structure figures were prepared using the PyMOL program (Schrödinger).

### ATPase assay

The ATPase activity assay was performed as previously described and slightly modified (Burgess et al., 2016b). Briefly, ATPase reaction mixture (50 μl) contained 20 mM Tris-HCl (pH 8), 5 mM DTT,10 mM MgCl_2_, 0.5 μCi(~300 nM) [γ-^32^P] labeled ATP and 1 mM nonradioactive ATP. The mixture was incubated at 22°C. The reaction was initiated by the addition of 3.4 μM of enzyme. Samples (2 μl) were taken from reaction mixture and mixed with 2 μl of quenching buffer (0.2 M EDTA) stop the reaction. A single time point activity assay was used to compare wild-type (WT) Spa47 and its mutants. The reactions time was held constant for 4 min before quenching reactions. the resulting mixture were resolved by thin-layer chromatography (TLC) using polyethyleneimine-cellulose plate (Sigma). The running buffer contains 0.8 M acetic acids and 0.8 M LiCl. The plate was visualized and quantified using Typhoon Trio Variable Mode Imager (GE healthcare). A multiple time point activity assay was used to determine wild type Spa47 enzyme kinetics and was performed under similar conditions to the single time point assay. The ATP hydrolysis rate was measured as the amount of released Pi (in pmolar) per minute per μg of enzyme.

### Isothermal titration calorimetry

An isothermal titration calorimetry (ITC) assay was performed with a MicroCalTM iTC200 calorimeter (MicroCal, USA) at 25°C. Both the protein and the ATP homolog were dissolved in the same buffer (20 mM Tris-HCl, pH = 8.0, 100 mM NaCl, 5 mM MgCl_2_). The concentrations of Spa47Δ1-83 and the mutants were between 0.03 and 0.04 mM. The concentration of ATP analogs was between 1 and 2 mM. Titration consisted of 18 consecutive 2-μl injections of ATP analogs with a 120-s interval between injections using a stir rate of 600 rpm. The dilution heat of the ligand was measured by adding ligand to a buffer solution under these conditions, and the injection schedule was identical to that used for the protein sample. For titration of the protein with ATP, MgCl_2_ was omitted in the buffer. Data acquisition and analysis were performed using Origin software. A single binding site model was used for nonlinear curve fitting. ITC experiments were repeated twice for each ligand.

### Size-exclusion chromatography

A Superdex 75 10/300GL column (GE healthcare) was equilibrated with buffer containing 20 mM Tris-HCl (pH 8.0) and 100 mM NaCl and calibrated using molecular weight standards, γ-globulin (158 kDa), ovalbumin (45 kDa), myoglobin (17 kDa), and vitamin B12 (1.35 kDa). Purified Spa47Δ1-83 were loaded onto the column at a flow rate of 0.15 ml/min.

### Statistical analysis

Statistical analyses were performed using unpaired, one-tailed, Student's *t*-test in the ITC assay and one-way ANOVA analyses in the ATPase assays. The *p*-value < 0.05 was considered statistically significant. These tests were performed with GraphPad Prism 6.0 software (GraphPad Software, Inc., San Diego, CA, USA).

## Results

### Nucleotide affinity of Spa47 ATPase

**Figure 1:**
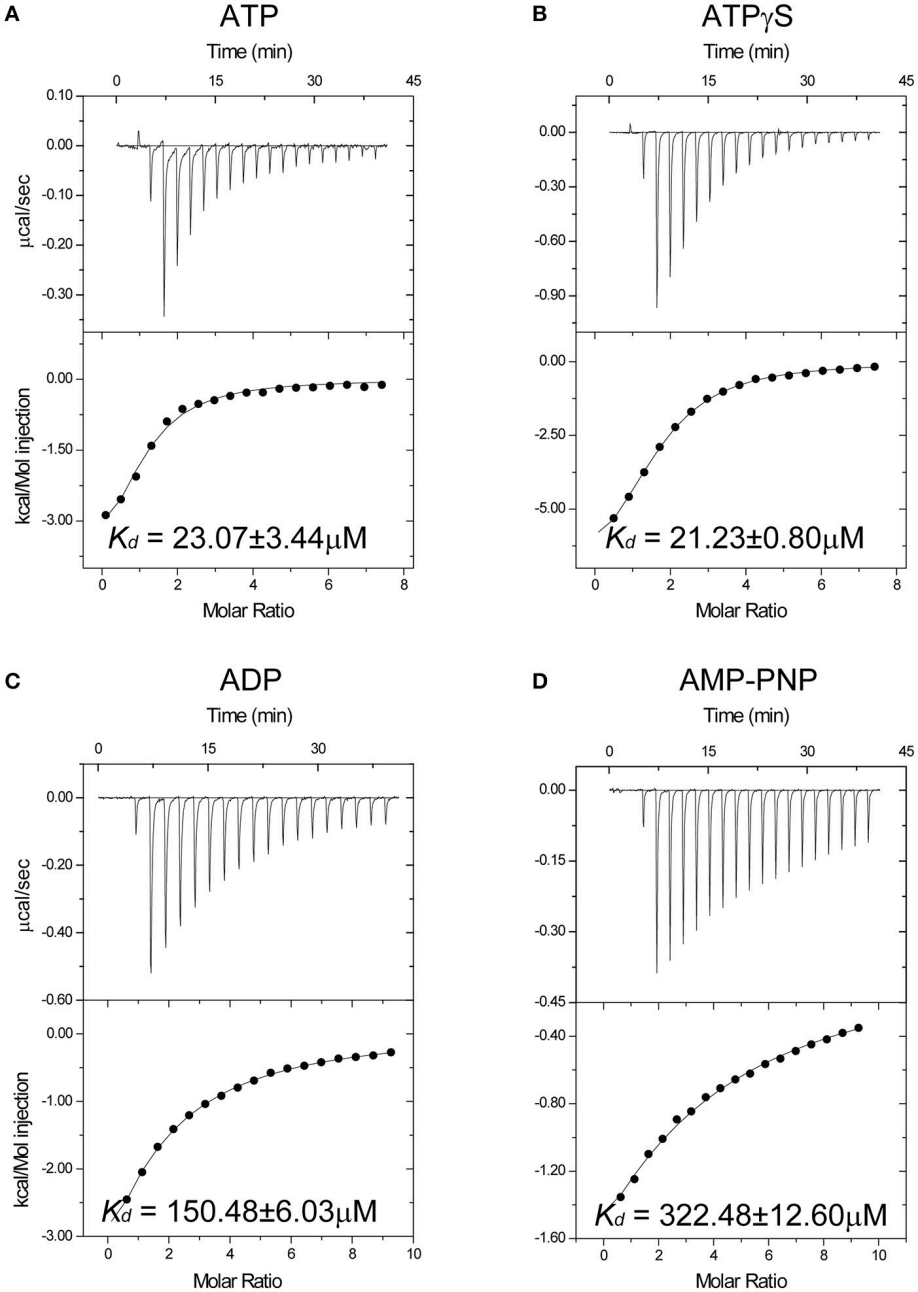
Isothermal titration calorimetry reveals nucleotide affinity of the Spa47 monomer. Titration curves (upper panels) and binding isotherms (lower panels) for monomeric Spa47Δ1-83 binding to **(A)** ATP without Mg^2+^, **(B)** ATPγS, **(C)** ADP, and **(D)** AMPPNP. The calculated *K*_*d*_ values are indicated in the lower panels. *K*_*d*_ values were calculated from single measurements, and errors were estimated by curve fitting.

Although many crystal structures of T3SS ATPases have been determined (Imada et al., 2007; Zarivach et al., 2007; Walker, 2013; Allison et al., 2014; Burgess et al., 2016a), including recently solved the structure of the homodimer of a C-terminal fragment of FliH (FliH_C2_) in complex with FliI (Imada et al., 2016). However, the predicted hexamer remains elusive. The complete ATP-binding pocket of T3SS ATPases is believed to be formed from two adjacent protomers within a hexamer. Thus, the monomer only harbors a partial ATP-binding pocket. This is supported by crystallographic studies of EscN and FliI ATPases in which AMPPNP and ADP are present at low occupancy in the ATP-binding pocket (Imada et al., 2007; Zarivach et al., 2007), and ADP is clearly visualized at high occupancy in the ATP-binding pocket of FliH_C2_-FliI complex(Imada et al., 2016). However, the monomeric truncated FliIΔ1–7, full-length Spa47 monomer, Spa47Δ1–6 monomer, SsaNΔ1–89 and other monomeric T3SS ATPases possess ATPase activity, although the monomeric Spa47Δ1–79 shows no ATPase activity (Minamino et al., 2006; Allison et al., 2014; Burgess et al., 2016a,b; Case and Dickenson, 2018). These results suggest that monomeric ATPases must have minimal capability to bind ATP that is sufficient to allow ATP hydrolysis. To elucidate the basis of ATP recognition, we performed isothermal titration calorimetry (ITC) experiments to investigate the nucleotide affinity of Spa47 ATPase from *S. flexneri*. Although full-length Spa47 is highly soluble (Burgess et al., 2016b), as shown in Figure S4A, it tended to precipitate during stirring in the sample cell of the ITC instrument, and was therefore not suitable for ITC experiments. We performed a screen for truncations and eventually identified a variant lacking the first 83 N-terminal residues (denoted Spa47Δ1-83). Spa47Δ1-83 was highly soluble and eluted from the size-exclusion chromatography column as a monomer (Figure S1). ITC experiments showed that Spa47Δ1-83 binds ATP with an apparent dissociation constant (*K*_*d*_) of 23.07 ± 3.44 μM, demonstrating that the Spa47Δ1-83 monomer can bind ATP (Figure 1A and Table S1) with comparable affinity to other ATPases/kinases. For example, muscle creatine kinase binds ATP with a *K*_*d*_ of 4–16 μM (Forstner et al., 1999), subunit B of the A_1_A_0_ ATP synthase binds ATP with a *K*_*d*_ of 22 μM (Kumar et al., 2009) and p97 binds ATP with a *K*_*d*_ of 0.89 μM (Tang et al., 2010). Next, we measured the binding affinity of two commonly used ATP analogs, ATPγS and AMPPNP, and found that Spa47Δ1-83 exhibited an evident preference for binding ATPγS over AMPPNP (Figures 1B,D). The binding affinity measured for ATPγS was similar to that for ATP (*K*_*d*_ = 21.23 ± 0.80 μM). The *p*-value for ATP vs. ATPγS is 0.23 (95% confidence interval [CI], −10.97 to 16.99). There are no significant differences between the binding of ATP and ATPγS to Spa47Δ1-83. By contrast, the binding affinity for AMPPNP was significantly lower (*K*_*d*_ = 322.48 ± 12.60 μM), ~15-fold lower than the affinity measured for ATP and ATPγS. The binding affinity for AMPPNP was even lower than the affinity for the product of ATP hydrolysis, ADP (*K*_*d*_ = 150.48 ± 6.03 μM; Figure 1C). The *p*-values for ADP vs. ATP and AMPPNP vs. ATP are 0.03 (95%CI, −6.65 to 336.00) and 0.02 (95%CI, 55.91–678.60), respectively. Thus, there are significant differences between these two ATP analogs and ATP in terms of binding to Spa47Δ1-83.

### Overall structure of Spa47Δ1-83

**Figure 2:**
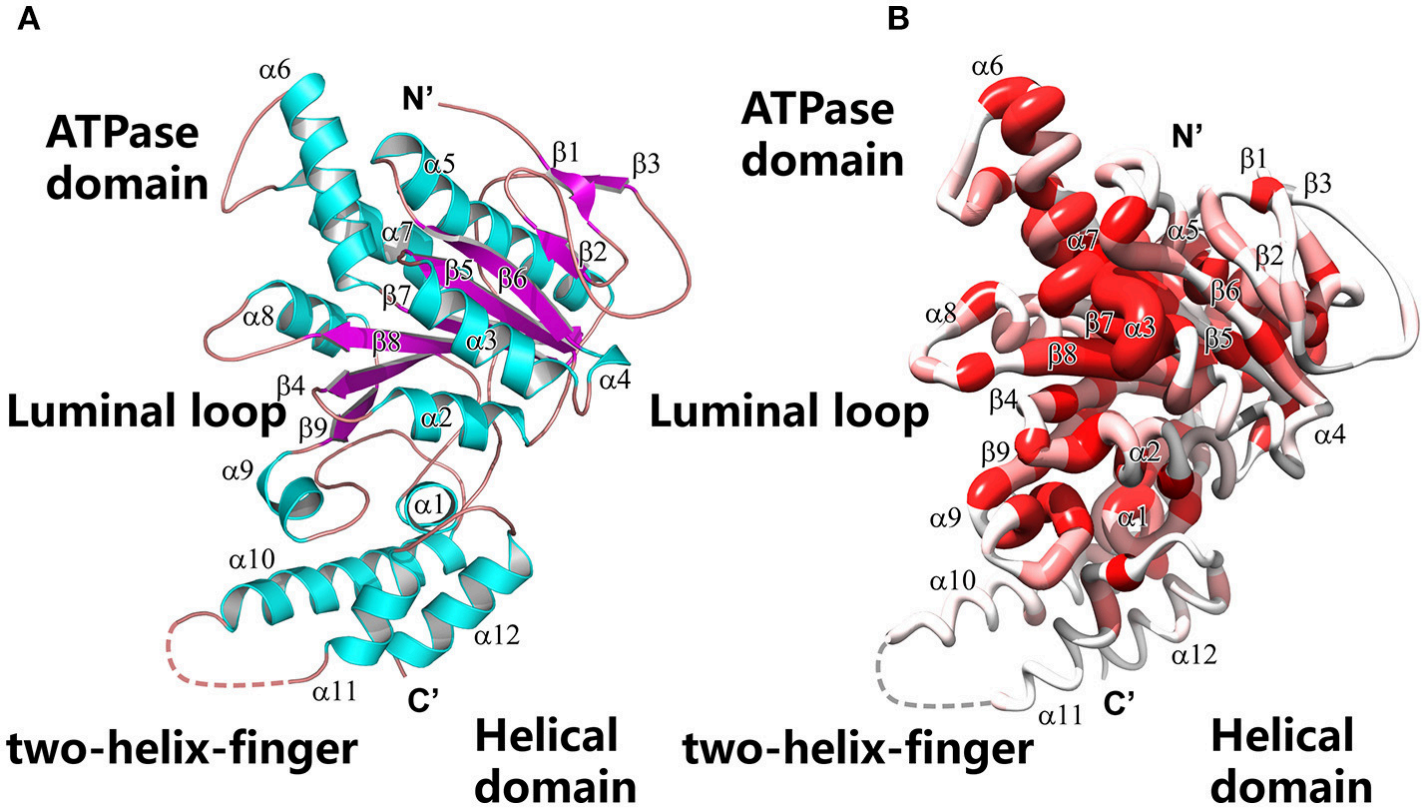
Overall structure of the Spa47 monomer. **(A)** Ribbon model of Spa47Δ1-83. Secondary structural elements are colored differently (α-helices are cyan, β-sheets are magenta and loops are ruby) and labeled. Key structural features are indicated. **(B)** Worm model of Spa47Δ1-83 in the same orientation as **(A)**, created using Chimera (Pettersen et al., 2004). The local thickness of the worm is proportional to the sequence conservation and colored in a gradient from red (invariant residues) to white (variable residues). Sequence conservation was calculated based on the multiple sequence alignment shown in Figure S2.

To reveal the structural basis of nucleoside triphosphate recognition, we determined the crystal structures of unliganded Spa47Δ1-83 and nucleotide-bound Spa47. The final atomic models yielded excellent refinement parameters and stereochemical quality (Table 1). In the unliganded structure, two Spa47 monomers are present in the asymmetric unit (ASU). Using PDBePISA software to analyse the crystal structure of Spa47Δ1-83, we did not observe an oligomeric assembly, consistent with the results of size-exclusion chromatography (Figure S1). The overall structure of Spa47Δ1-83 is similar to that of other T3SS ATPases, including the monomeric Spa47Δ1–79 N-terminal truncation (Burgess et al., 2016a). It includes a conserved ATPase domain spanning residues 84–354, and a C-terminal helical bundle domain spanning residues 355–430 (Figure 2A). The ATPase domain exhibits an α/β Rossmann fold comprising a twisted plane of nine parallel β-strands that is sandwiched by seven α-helices on both sides. The Walker A motif spans residues 159–165, forming a phosphate-binding loop (P-loop) between β4 and α2. The Walker B motif is located between β7 and α6, and includes a negatively charged residue (D249) involved in magnesium coordination (Figure S2). The C-terminal helical domain comprises helices α10–12, wrapped into a distorted helical bundle. The loop connecting α10 and α11 is missing in the electron density map, reflecting an intrinsic flexibility in this region. We generated a worm model of the Spa47Δ1-83 structure, in which the thickness and coloring are correlated to sequence conservation (Figure 2B). The model shows that, while the central ATPase domain is highly conserved, the C-terminal helical bundle is more variable, consistent with the role of the helical domain in the recognition of effector proteins.

**Table 1:** Data collection and refinement statistics.

	**Spa47 (84–430) (PDBID: 5YBH)**	**Spa47 (84–430) and ATPγS (PDB ID: 5ZT1)**	**Spa47 (84–430) and AMPPNP (PDB ID: 5YBI)**
**DATA COLLECTION**			
Space group	P 32 2 1	P 32 2 1	P 32 2 1
**Cell dimensions**			
a, b, c (Å)	105.38, 105.38, 146.38	104.26, 104.26, 145.76	105.01,105.01,146.64
α, β, γ (°)	90.00, 90.00, 120.00	90.00, 90.00,120.00	90.00, 90.00,120.00
X-ray source	SSRF BEAMLINE BL17U	SSRF BEAMLINE BL17U	SSRF BEAMLINE BL17U
Wavelength (Å)	0.9792	0.9792	0.9792
Data range (Å)	35.0–2.50	30–3.10	45.0–2.27
Reflections unique	32,716	31,772	82,611
*R*_sym_^a^ (last shell)	0.109 (0.684)	0.168 (0.746)	0.092 (0.613)
*I*/σ*I* (last shell)	25.38 (5.60)	17.78 (5.23)	20.56 (4.27)
Completeness (%) (last shell)	97.9 (96.6)	99.3 (96.5)	98.5 (98.8)
Redundancy (last shell)	16.4 (15.6)	9.43 (8.86)	8.52 (8.44)
**REFINEMENT**			
Resolution range (Å)	34.50–2.49	29.86–3.11	43.43–2.27
Reflections (non-anomalous), cut-off, cross validation	33,404 (32,714), *F* > 1.35, 1,653	31,537 (16,817), *F* > 1.34, 3,127	83,839 (82,596), *F* > 1.34, 4,138
*R*_work_^b^/*R*_free_^c^ (last shell)	0.1917/0.2480 (0.2430/0.3069)	0.1915/0.2546 (0.3301/0.3480)	0.1794/0.2270 (0.2763/0.3046)
**ATOMS**			
Non-hydrogen protein atoms	10,666	10,525	11,283
Protein	10,422	10,412	10,814
Mg ion	20	29	38
Nucleotide	0	45	47
Sulfate ion	25	10	35
Solvent	199	29	349
*B*-factor average (Å^2^)	50.82	46.21	42.52
Protein (Å^2^)	50.90	46.11	42.42
Ion (Å^2^)	54.83	53.94	55.82
Ligand (Å^2^)		58.99	60.66
Water (Å^2^)	45.40	60.52	40.22
**r.m.s.d**			
Bond lengths (Å)	0.009	0.017	0.010
Bond angles (°)	1.135	1.466	1.149

a *R_sym_ = ∑_hkl_∑_j_ |I_hkl, j_ – I_hkl_|/∑_hkl_∑_j_I_hkl, j_, where I_hkl_ is the average of symmetry-related observations for a unique reflection*.

b *R_work_ = ∑_hkl_||F_obs_(hkl)|–|F_calc_(hkl)||/∑_hkl_|F_obs_(hkl)|*.

c *R_free_ = cross-validation R factor for 5% of reflections, against which the model was not refined*.

### Crystal structures of nucleotide-bound Spa47Δ1-83

**Figure 3:**
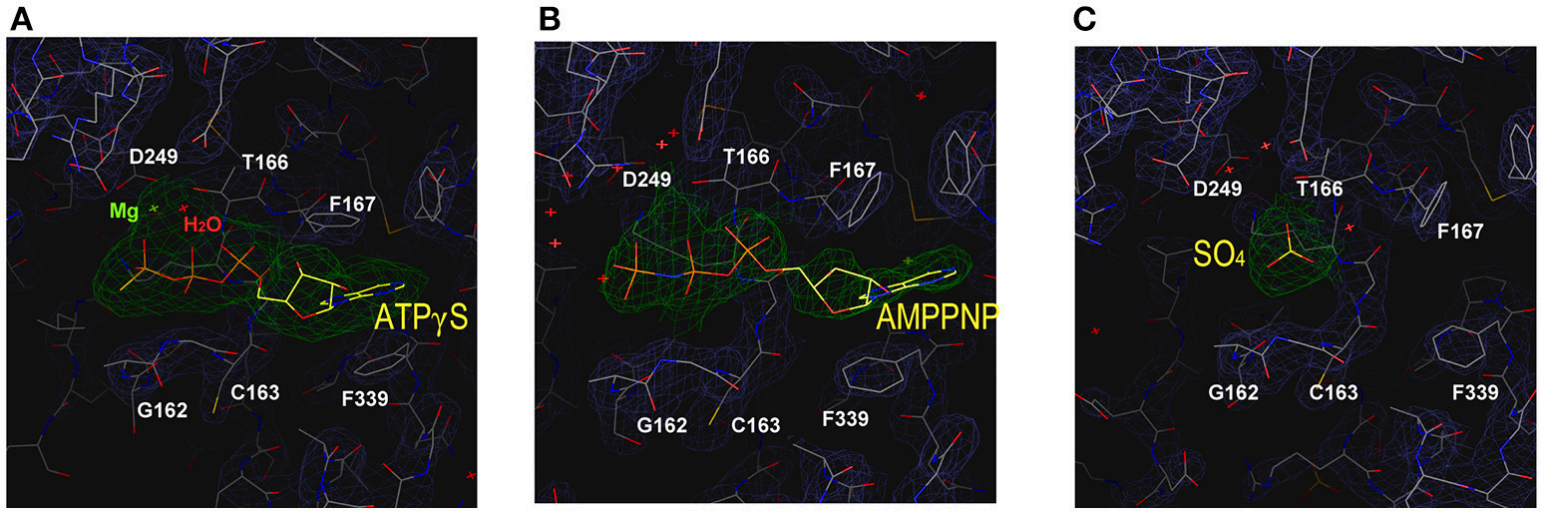
Structure of the ATP-binding site in the various enzyme-ligand complexes. The magnified views show the ATP-binding site of Spa47 bound to **(A)** ATPγS, **(B)** AMPPNP, and **(C)** the empty ATP-binding site without ligand soaking. The sulfate at the P-loop is derived from the crystallization buffer. Residues are shown in stick representation, and the final electron density map (2Fo-Fc, contour at 0.8 σ) is superimposed. Residues involved in nucleotide recognition are indicated.

We performed crystal soaking experiments to determine the crystal structures of Spa47Δ1-83-ATPγS and Spa47Δ1-83-AMPPNP. These crystal structures were solved by molecular replacement using the unliganded Spa47Δ1-83 structure as the search model. As shown in Figure 3A, we observed the electron density for the entire ATPγS molecule, a catalytically important Mg^2+^ and an ordered water molecule at the P-loop. Because ATPγS has a γ-thio substitution at its γ-phosphate group, we located the sulfur atom in the final electron density map by fitting it into the larger-than-usual (i.e., sulfur rather than oxygen) density peak. The length of the phosphorus-sulfur bond was ~2.0 Å, consistent with a typical P-S single bond. There were no interactions found between the sulfur and the protein. In stark contrast, we observed much less electron density for AMPPNP at the P-loop (Figure 3B). Indeed, only the triphosphate moiety could be located in the electron density map, and electron density for the sugar and adenosine base was poor. In the final refined model, the occupancy for AMPPNP is < 1.0. To make binding of AMPPNP more favorable, we increased the concentration of AMPPNP in the crystal soaking experiment. However, neither the electron density nor the occupancy of AMPPNP could be improved, even in the presence of 85 mM AMPPNP. When comparing the structures of unliganded Spa47Δ1-83 and the nucleotide-bound enzyme, we found that solvent molecules occupying the P-loop could be expelled upon nucleotide binding. In the unliganded Spa47 structure, a number of water molecules, and a ${\text{SO}}_{4}^{2-}$ ion occupy the P-loop (Figure 3C). However, when ATPγS accommodates the active site of Spa47Δ1-83, all solvent molecules are expelled except the catalytic magnesium and one ordered water molecule (Figure 3A). However, this was not the case in the Spa47Δ1-83-AMPPNP structure; the ATP-binding site remained occupied by a number of solvent molecules (Figure 3B), resembling the situation in the unliganded structure.

### Determinants of ATP recognition

**Figure 4:**
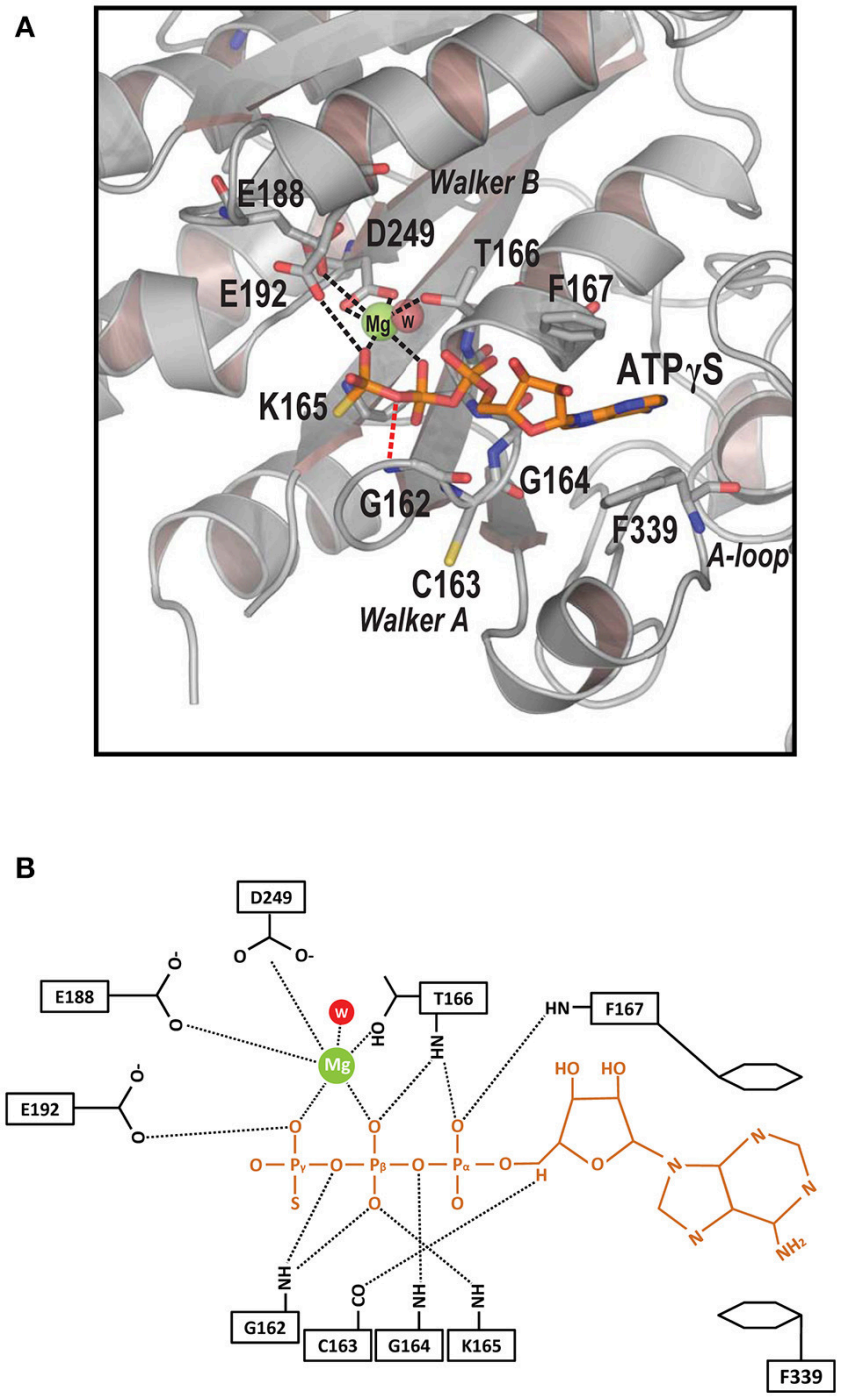
Determinants of ATP recognition in Spa47. **(A)** Ribbon model of the ATP-binding pocket (gray) with bound ATPγS (orange), catalytic Mg^2+^ (green), and an ordered water molecule (red). Residues recognizing nucleotides and magnesium are shown in stick representation. Contacts involved in Mg^2+^ recognition and stabilization are indicated by black dashed lines. A specific hydrogen bond recognizing the oxygen between the γ- and β-phosphates is indicated by a red dashed line. Key structural features are indicated. **(B)** Detailed diagram of interactions between the ligand ATPγS (orange), catalytic Mg^2+^ (green), the ordered water molecule (red), and Spa47 (black). Interactions are marked with dashed lines.

The structure of the Spa47Δ1-83-Mg-ATPγS complex facilitates a complete description of nucleoside triphosphate recognition by the Spa47 monomer at an atomic level (Figure 4A). The catalytic magnesium is coordinated by the β- and γ-phosphates of ATPγS, the aspartate side chain of D249 from the Walker B motif, the glutamate side chain of E188 and the OH group of T166, suggesting that these residues participate in ATP hydrolysis. An ordered water molecule is located within hydrogen bonding range (2.7 Å) of the catalytic Mg^2+^ (Figures 4A,B). The Walker A motif (^162^GCGKT^166^) forms the P-loop that binds the triphosphate moiety of ATPγS through multiple hydrogen bonds (Figure 4B). Residue E192 forms an additional hydrogen bond with the γ-phosphate of ATPγS. Of these bonds, the hydrogen bond between NH of G162 and the oxygen between the β- and γ-phosphates of ATPγS (length = 2.5 Å, angle = 130.4°) plays a vital role in ATP recognition; when this oxygen was replaced by an NH group in AMPPNP, the hydrogen bond donated by G162 was not observed (Figure 3B), and the loss of this hydrogen bond could explain the dramatic reduction in binding affinity for AMPPNP.

Electron density for the ribose moiety of ATPγS is relatively well ordered even though this portion protrudes from the protein (Figure 3A). The adenosine base of ATPγS is sandwiched between a pair of aromatic residues (F167 and F339). The side chain of F339 stacks against the base of ATPγS via π-π interactions. F167 further stabilizes the adenosine base by π-stacking against the opposite side of the base. Comparing the structures of unliganded and ATPγS-bound Spa47Δ1-83 (Figures 3A,C), we found that ATPγS binding induced evident rotation of the aromatic side chains F167 and F339 around the C_β_-C_γ_ axis, so that the benzyl rings remain parallel to the adenosine base to facilitate π-stacking. By contrast, a similar movement of the side chains of F167 and F339 was not observed in the Spa47Δ1-83-AMPPNP structure (Figure 3B), indicating that the base of AMPPNP did not fully occupy the ATP-binding pocket.

### Conformational changes in the luminal loop are associated with ATP binding

**Figure 5:**
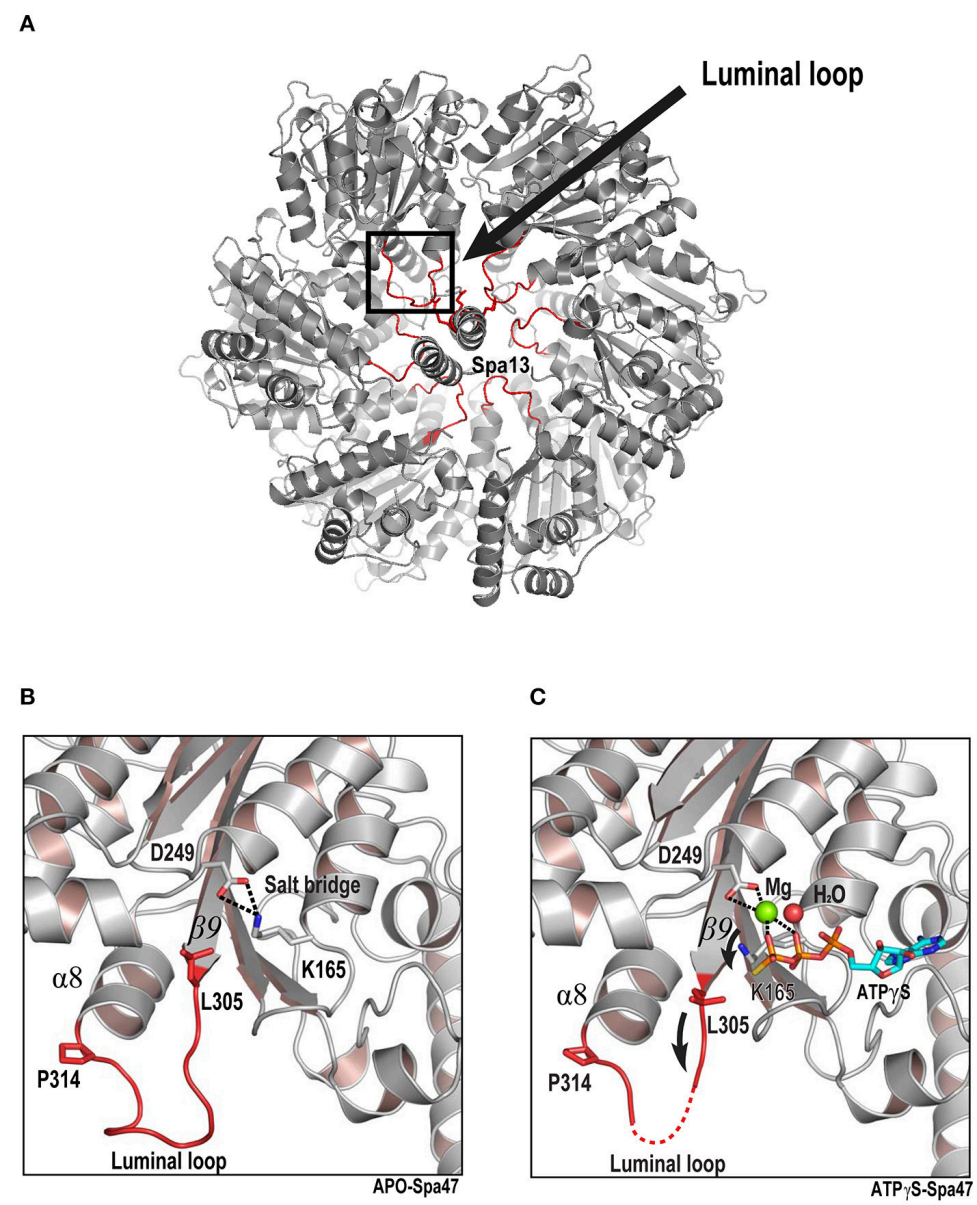
ATP binding triggers conformational changes that are transmitted from the ATP-binding site to the luminal loop. **(A)** Model of the proposed hexameric ring of Spa47 and Spa13 complex based on the F_1_ ATP synthase structure (PDB ID: 1BMF) as the template. The luminal loop (residues L305, L306, E307, D308, D309, D310, F311, A312, D313, and P314) are highlighted in red. Luminal loops project into the predicted pore of the hexamer. The RMSD value calculated from aligning the Spa47 structure onto the F1 ATPase hexamer is 1.9 Å. **(B,C)** Crystal structures of unliganded and ATPγS-bound Spa47. The ATP-binding site (gray) and luminal loop (red) are magnified. Residues undergoing conformational changes are shown in stick representation. Mg^2+^ (green) and water (red) are shown as spheres. Nucleotides are colored cyan for carbon, red for oxygen, blue for nitrogen and yellow for sulfur. A strong salt bridge between D249 and K165 is indicated by dashed lines. Black arrows indicate the direction of movement.

**Figure 6:**
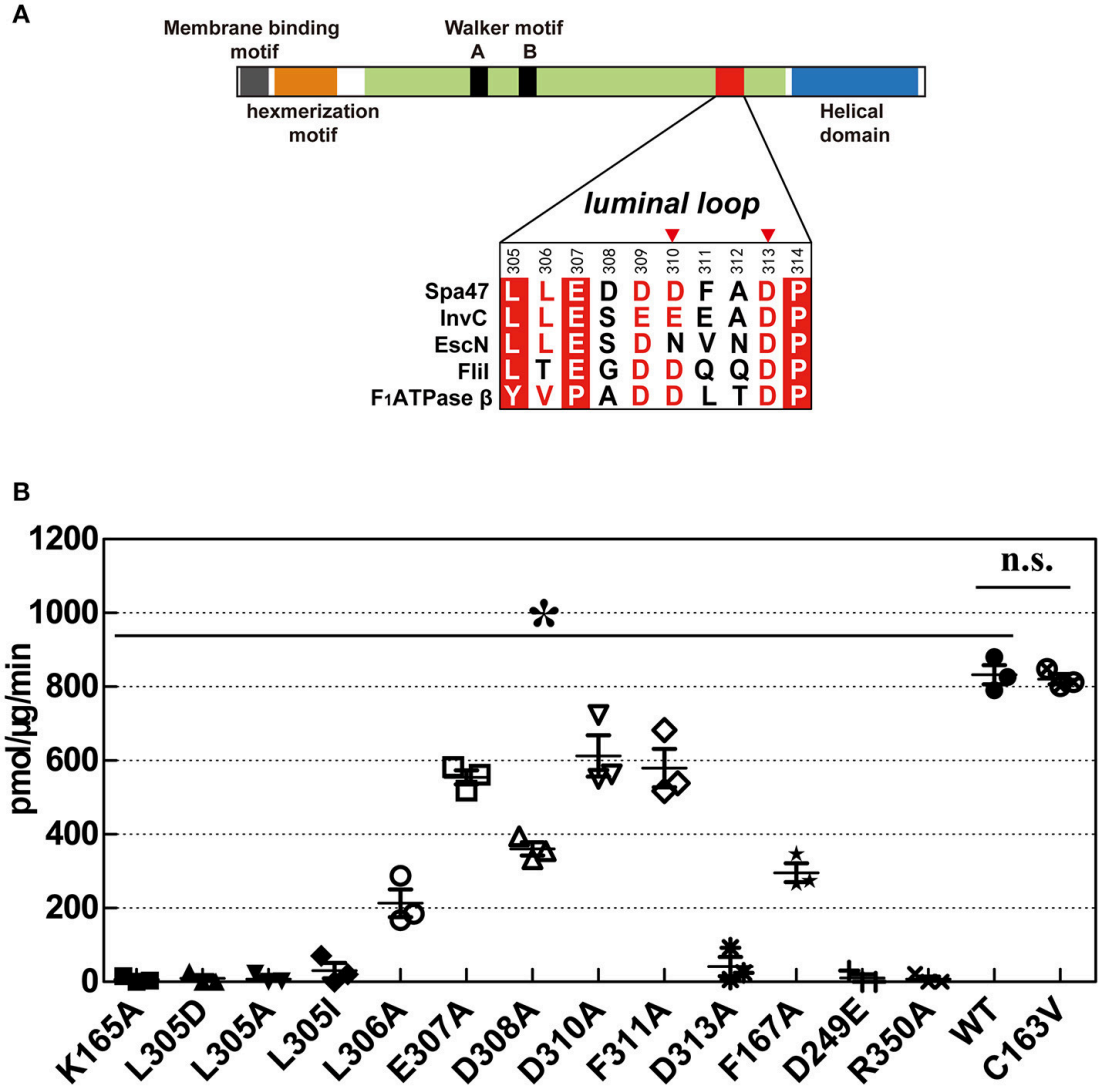
ATPase activity of wild-type Spa47 and various mutants. **(A)** Domain organization of Spa47. The sequence alignment includes residues from the luminal loop of type III ATPases Spa47, InvC, EscN, FliI, and F_1_ATPase β. Invariant residues are highlighted with a red background, conserved residues are colored red and variable residues are colored black. Red triangles mark residues that may be involved in interaction with Spa13. **(B)** Histogram of the ATPase activity of various mutants in which catalytically important residues are substituted. The inactive mutants K165A and R350A served as negative controls for ATPase activity. Results are averages of three independent measurements, and error bars represent calculated standard deviations. Asterisks represent statistically significant differences (one-way ANOVA analyses, *P* < 0.05) between WT and mutants, n.s., non-significant.

The structures of unliganded Spa47Δ1-83 and Spa47Δ1-83-ATPγS represent two different states. Comparing these two structures allowed us to gain insight into the conformational changes associated with ATP binding. We discovered that the conformation of the loop between β9 and α8 was dramatically affected following binding of ATPγS. The β9–α8 loop is a highly conserved region in T3SS ATPases and F_1_ATPase β subunit (**Figure 6A** and Figure S2). To reveal its position in the context of the proposed hexamer, we generated a hexameric ring model of Spa47Δ1-83 by superimposing monomers onto all subunits of the F_1_ATPase hexamer (PDB ID: 1BMF). The hexameric model shows that the β9–α8 loop projects into the center of the ring with Spa13 penetrating into the central hole of the Spa47 hexamer in a similar way to the γ subunit penetrating into the pore of the α_3_β_3_ ring in F_1_ATPase or FliJ in the pore of the FliI ATPase (Abrahams et al., 1994; Ibuki et al., 2011; Hu et al., 2015, 2017) (hence it is denoted the luminal loop) (Figure 5A). In the unliganded Spa47Δ1-83 structure, the side chains of the catalytically important residues K165 (Walker A motif from the P-loop) and D249 (Walker B motif) are connected by a salt bridge (Figure 5B). The luminal loop is located below the P-loop and exhibits relatively stable folding, as demonstrated by the observation of sufficient electron density for this region (Figure S3A), and the average B-factor of the loop (residues 305–315) was 81.9 Å^2^. In the Spa47Δ1-83-ATPγS structure, ATPγS, Mg^2+^ and an ordered water molecule accommodate the P-loop and occupy the space taken by the K165 side chain in the unliganded structure (Figure 4A). Consequently, the salt bridge between K165 and D249 in the unliganded structure is broken (Figure 5C), and the side chain of K165 is pushed ~3 Å in the direction of the luminal loop, where it clashes with the side chain of L305. Additionally, the side chain of L305 is also shifted toward the luminal loop. Collectively, our structural comparison shows that the binding of ATPγS and magnesium initiates conformational changes, which appear to spread from the P-loop to the downstream luminal loop. In addition, these conformational changes could increase the flexibility of the luminal loop, since the conformation of the luminal loop is more flexible in the Spa47Δ1-83-ATPγS structure than in the unliganded structure. Electron density for the luminal loop is reduced significantly or even missing in the Spa47Δ1-83-ATPγS structure (Figure S3B), and the average B-factor of the loop is significantly increased (~97.0 Å^2^). Several residues at the apex of the luminal loop could not be located. Combining these observations, disruption of the luminal loop appears to be caused by ligand binding during crystal soaking. ATPγS binds to the active site of Spa47, and their binding therefore alters the conformation of neighboring residues.

### Mutagenesis study

To validate our structural findings, we engineered a collection of full-length Spa47 mutants and tested their ATPase activity. Full-length Spa47 was fused at its N-terminus to a self-cleavable VMA1 intein-CBD tag (56 kDa), which facilitates affinity purification of the fusion precursor on a chitin column, followed by dithiothreitol (DTT)-induced self-cleavage of protein splicing elements (inteins) to separate Spa47 from the affinity tag (Burgess et al., 2016a,b; Figure S4A). Each of the mutations was introduced in the full-length Spa47 protein, and purification was as described above for native full-length Spa47. ATPase activity was measured using thin layer chromatography (TLC) to separate radioactively labeled free phosphate from the ATP substrate. Activity was calculated by dividing the amount of ATP-ADP converted by the reaction time and the amount of enzyme in the assay. The results showed that full-length Spa47 exhibited ATP hydrolysis activity with a *k*_cat_ of ~0.15 ± 0.001 s^−1^, which is similar to that reported previously for monomeric Spa47 (Burgess et al., 2016a,b; Figure S4B). In addition, the Spa47 K165A and R350A mutants lacking ATPase activity were included as negative controls to measure the background rate of ATP hydrolysis (Figure 6B and Figure S4C). The highly conserved R350 is responsible for ATP hydrolysis by the Spa47 ATPase in a way similar to the FliI ATPase although R350 does not contribute to the binding of ATPγS in a crystal of the ATPγS-bound form of the Spa47Δ1-83 (Kazetani et al., 2009), as also described previously (Burgess et al., 2016a,b).

The Walker A motif mutant C163V exhibited a similar rate of ATP hydrolysis to wild-type (WT) Spa47. Residue C163 is located between two invariant glycine residues in the Walker A motif (^162^GCGKT^166^). The side chain of C163 is buried in the hydrophobic core of Spa47, distant from the P-loop, explaining why the C163V substitution does not affect the conformation of the P-loop. Substitution of F167, which stabilizes the adenosine base of ATPγS, reduced the ATP hydrolysis rate, but a fraction of ATPase activity was retained.

Our crystallographic results demonstrated that ATP binding may cause conformational changes in the luminal loop located at the pore of the proposed Spa47 hexamer. A direct interaction between the side chains of K165 and L305 appears to be essential for ATP hydrolysis. L305 are highly conserved in T3SS ATPases (Figure 6A) and are essential for ATP hydrolysis, because mutations L305A, L305D, and L305I all led to abrogation of the ATPase activity of Spa47 (Figure 6B and Figure S4C). Additionally, substitution of residues at the apex of the luminal loop (residues 307–311) caused a slightly reduction in ATP hydrolysis rate, but ATPase activity was still retained. Alanine scanning mutagenesis was performed between residues E307 and F311, and these mutants exhibited ~43–74% of the ATPase activity of WT Spa47 (Figure 6B and Figure S4C). However, ATPase activity was particularly affected in the D313A mutant. This residue is located near the interface of the Spa47 protomers in the proposed hexamer, and a previous study showed that this mutation most likely affects the cooperative interaction of the protomers that is required for efficient ATP hydrolysis (Kazetani et al., 2009; Burgess et al., 2016a), as was also concluded from an equivalent mutation of D312 in the *Salmonella* SPI-1 ATPase InvC (Kato et al., 2015).

## Discussion

The T3SS ATPase utilizes energy from ATP hydrolysis to separate chaperone proteins from effector proteins, unfold effector proteins and eventually force them through the central channel of the secretion apparatus (Akeda and Galan, 2005; Galán, 2008). In many ways, the function of T3SS ATPase is similar to the unfoldase and translocase activities of AAA+ family members. Kato and colleagues identified a two-helix-finger motif and a luminal loop located at the central pore in the predicted hexameric ring of InvC (Kato et al., 2015) that is reminiscent of similar features in ATP-powered unfoldase and translocase enzymes. Based on structural modeling, they predicted that ATP hydrolysis leads to conformational changes in the two-helix-finger motif and the luminal loop. Through this mechanism, movement of the two-helix-finger motif and the luminal loop may propel the effector protein through the pore of the hexamer, thus facilitating effector secretion. However, Ibuki and colleagues showed that the two-helix-finger motif of the FliI ATPase is responsible for the interaction with the FliJ by pull-down assays *in vitro* (Ibuki et al., 2011), comparable with the loop between helices 1 and 2 located in the C-terminal region of the β subunit acting as the binding region for the γ subunit in F_1_ATPase (Abrahams et al., 1994). Furthermore, FliI does not induce the release of flagellar chaperone from the chaperone-substrate complex in an ATP-dependent manner (Minamino et al., 2012). Consistently, the InvC ATPase is not essential for protein secretion when the PMF is sufficiently high (Erhardt et al., 2014). In the present study, we also identified a luminal loop located at the central pore in the predicted hexameric ring of Spa47 ATPase, and showed that the conformational changes of the luminal loop is induced by ATP binding. Interestingly, this luminal loop is highly conserved (Figure 6A), in the F_1_ATPase structure, the corresponding region of the β subunit is the portion with which the γ subunit interacts (Abrahams et al., 1994). These observations suggest that Spa47 ATPase likely interacts with Spa13 (a FliJ/γ subunit homolog) in a similar way to the γ subunit interacting with the β subunit in F_1_ATPase.

Therefore, the precise mechanistic role that T3SS ATPase provides to support secretion or protein export is clearly complex and remains somewhat controversial. It is tempting to speculate that ATP hydrolysis by T3SS ATPase provides energy for protein secretion. However, the well-studied bacterial flagellum type III secretion system (f-T3SS) provides novel insight into T3SS ATPase function in protein export, since it utilizes both ATP hydrolysis and the PMF as energy sources for the translocation of substrates across biological membranes(Minamino and Namba, 2008; Paul et al., 2008; Erhardt et al., 2014; Lee et al., 2014). Consistently, FliH, FliI, and FliJ are dispensable for flagellar protein export, and the PMF is the main power source for unfolding and translocation of export substrates (Minamino and Namba, 2008; Paul et al., 2008). Additionally, it has been shown that the FliI ATPase with the E211D/E211Q mutation resulting in infrequent ATP hydrolysis or no ATP hydrolysis is enough for processive flagellar protein export, suggesting that the FliI ATPase seems to act as a export gate activator (Minamino et al., 2014). In the presence of FliH, FliI, and FliJ, interaction between FliJ and an export gate membrane protein FlhA is supported by the FliH-FliI complex, which fully activates the export gate, turning it into an efficient, PMF-driven protein export apparatus (Minamino et al., 2011; Lee et al., 2014). Interestingly, a similar result was recently reported for T3SS from *Shigella* and *Salmonella* (Cherradi et al., 2014; Erhardt et al., 2014), and Morimoto and colleagues showed that ATP hydrolysis by FliI and subsequent rapid protein translocation are both linked to proton translocation through the export gate (Morimoto et al., 2016). These observations indicate that the flagellar transmembrane export gate acts as a PMF-driven translocase or unfoldase, and the FliI ATPase does not engage in unfoldase activity but ensures that substrates are export competent (Minamino et al., 2016).

The recent high-resolution *in situ* structure of the *Salmonella* T3SS machine obtained by cryo-electron tomography(cryo-ET) and sub-tomogram averaging provides further insight into T3SS ATPase function. Hu and colleagues showed that InvI/Spa13 is located in the central pore of the T3SS ATPase, as also shown for FliJ(a Spa13 homolog), also binds to the central pore of the FliI ATPase hexamer (Ibuki et al., 2011; Hu et al., 2015, 2017). Therefore, the previous model in which T3SS ATPase unfolds substrates by “threading them” through the center of the hexameric channel is probably untenable because the central pore of the hexameric ATPase is blocked by the central stalk protein [FliJ in the f-T3SS and SctO in the virulence T3SS (v-T333)], thereby preventing export of the substrates. In addition, the Spa33 pods structure are linked by a six-spoke wheel-like structure(MxiN) with a central Spa47 hexamer structure, thus capping the central channel, also preventing the translocation of the substrates (Hu et al., 2015). Finally, the C-terminal region of the T3SS ATPase is oriented toward the proximal side of the export apparatus, similar to F/V-type ATPase (Imada et al., 2016; Hu et al., 2017). The C-terminus is predicted to be a docking site for substrate or chaperone (Allison et al., 2014). Therefore, it can be inferred that the central channel of the homo-hexameric ring is not involved in substrate translocation, otherwise the substrate could not reach the export gate, and would instead be directed backwards, away from the export gate. Therefore, the model in which ATP-driven conformational changes of the conserved luminal loop in T3SS ATPase are coupled to substrate recognition, unfolding, and translocation through the center of the hexameric channel is controversial (Kato et al., 2015). Analysis of the available data suggests that the v-T3SS ATPase first unfolds and removes the type III secretion substrates from their cognate chaperones via ATP hydrolysis, and then initiates entry of substrate into the secretion pathway via mechanisms that are not yet fully understood but most likely involve the PMF. Thus, ensuring that substrates are export-competent may be the primary function of the ATPase (Lee and Rietsch, 2015).

**Figure 7:**
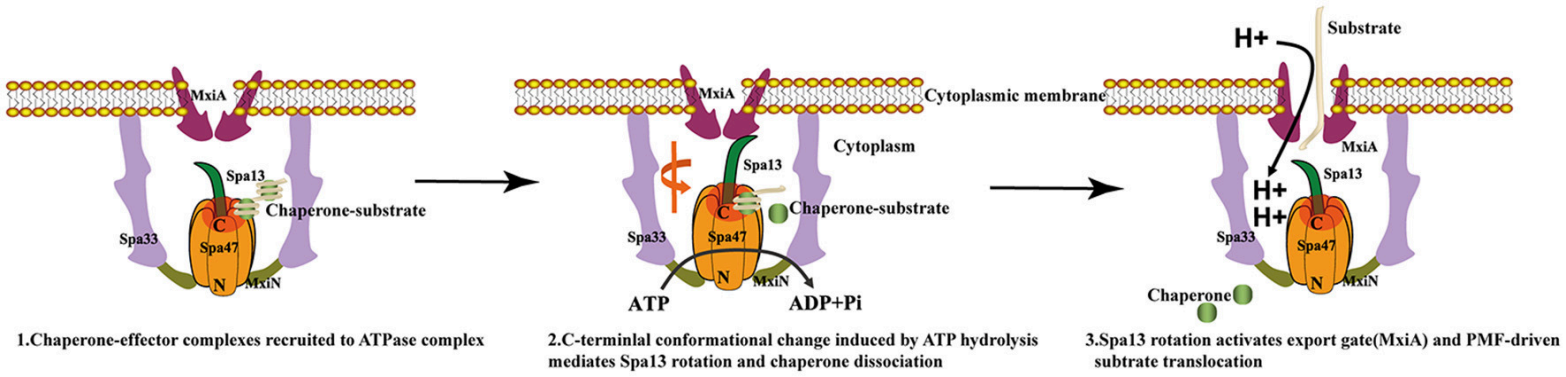
Models of T3SS protein export. (1) Chaperone-substrate complexes are recruited to the cytosolic ATPase complex; (2) ATP hydrolysis by the Spa47 hexamer induces a conformational change in the C-terminal region of Spa47 that is coupled to the Spa13 rotation within the ring and chaperone dissociation; (3) Spa13 rotation activates the export gate (MxiA) and then the export gate utilizes the proton motive force across the cytoplasmic membrane to transport the substrates into the T3SS channel.

Since the structures of the f-T3SS and v-T3SS share a similar cytoplasmic ATPase ring complex consisting of the homo-hexameric ATPase and a central rod structure, similar to proton-translocating F/V-type rotary ATPases, they probably also share a similar mechanism. It has been shown that the f-T3SS acts as a proton-protein antiporter in a similar way to F_1_F_0_ ATPase, which both hydrolyze ATP to induce an outward-directed proton pumping (Morimoto et al., 2016). The well-characterized F/V-type ATPase couples ATP synthesis and hydrolysis to proton translocation across the membrane, and the F-type ATPase is a rotary motor driven by sequential ATP binding and hydrolysis at three catalytic sites in the α_3_β_3_ ring (Abrahams et al., 1994; Mulkidjanian et al., 2007), this ATP binding and hydrolysis is coupled with the conformational changes in the relative orientations of the ATPase and C-terminal domains of the β subunits with the rotation of the γ subunit in the middle of the ring (Menz et al., 2001; Imada et al., 2007). Our structural investigation of Spa47 from *S. flexneri* provides experimental evidence supporting this hypothesis. Piecing together the structures of Spa47-ATPγS and unliganded Spa47 facilitated the identification of a highly conserved luminal loop, the luminal loop contributes to efficient ATP hydrolysis by the Spa47 ATPase (Figures 5, 6). Our crystallographic results showed that binding of ATPγS and Mg^2+^ breaks the salt bridge between D249 and K165 and shifts the side chain of K165 by approximately 3 Å. The shift in the side chain of K165 is accommodated by the movement of L305 (Figure 5C), this movement is coupled with conformational changes of the luminal loop. The luminal loop is highly conserved, and residues D310 and D313 form hydrogen bonds with γ subunit residues R254 and Q255 in F_1_ATPase (Abrahams et al., 1994; Figure 6A). Therefore, we propose that a conformational change of a conserved luminal loop in Spa47 accompanied by ATP binding, which is most likely linked to the rotation of Spa13, similar to rotation of the γ subunit in F_1_ATPase. The type III protein export apparatus is postulated to act as a proton-protein antiporter to couple ATP hydrolysis with the proton flow to drive protein export (Figure 7). This model is also supported by evidence from f-T3SS and v-T3SS that cytoplasmic component FliJ/InvI(YscO), the homolog of Spa13, is visualized to be located in the central pore of the ATPase hexamer ring and involved in controlling the conversion of the PMF to protein export (Hara et al., 2011; Minamino et al., 2011; Lee et al., 2014; Hu et al., 2015, 2017; Lee and Rietsch, 2015), and Spa13 acts as an export-gate activator (Cherradi et al., 2014). However, direct evidence for this model is still lacking, and exactly how Spa13 activates the export gate remains unclear, although we speculate that ATP hydrolysis coupled with the rotation of Spa13 is likely involved. More work is also needed to understand how Spa47 interacts with Spa13, and the structure of the Spa47 hexamer in complex with Spa13 would undoubtedly help us to understand its role in the dynamics of the secretion process.

The luminal loop contains the conserved L305 residue, and a negatively charged motif. Alanine substitution of the negatively charged residues did not abrogate the ATPase activity of InvC *in vitro*, but it abolished secretion of the late substrates SptP and SopB (Kato et al., 2015). In the case of Spa47, neutralization of the negatively charged residues of the luminal loop does not severely diminish ATPase activity. However, alteration of L305 abolished the ATPase activity of Spa47 completely, suggesting that L305 (and presumably L306) are indispensable for the ATP hydrolysis-powered engine. In particular, L305 is intolerant to any tested substitution, including the conservative leucine to isoleucine mutation. These results suggest that highly specific side chain interactions between K165 and L305 are required for ATP hydrolysis.

ATP analogs trap ATPases in the nucleoside triphosphate-bound state, and can provide great insight into ATP recognition and hydrolysis. ATPγS and AMPPNP are commonly used ATP analogs in biochemical and structural investigations. A ligand search in the protein data bank found 116 structures containing ATPγS and 588 structures containing AMPPNP, showing that AMPPNP is more frequently used. However, differences between the two analog deserve to be emphasized. A complete ATPase-binding pocket is formed between two RecA-like domains in the SF2 helicase, and between adjacent protomers in the hexamer of the AAA+ ATPase and F-type ATPase (Walker, 2013). In these cases, ATPγS and AMPPNP may not exhibit differences in binding due to space constraints. By contrast, Spa47 presents a special case in which the monomer efficiently binds ATP. To our surprise, the T3SS ATPase monomer exhibited striking differences in the recognition of ATPγS and AMPPNP that have not been previously reported. In ITC experiments, ATPγS exhibited similar binding affinity to Spa47 as did ATP, but AMPPNP bound poorly to Spa47, with ~15-fold lower affinity. The crystal structure of Spa47-ATPγS facilitated a complete description of nucleoside triphosphate recognition by a T3SS ATPase (Figure 4). However, Spa47-AMPPNP structures failed to provide such deep insight, since AMPPNP was not recognized in the same manner. Similar results have been reported in a structural investigation of EscN, in which electron density for the γ-phosphate of AMPPNP was missing (Zarivach et al., 2007), and the salt bridge between D266 and K183 (corresponding to the salt bridge between D249 and K165 in Spa47) was unaffected, resembling the structure of the unliganded Spa47. Collectively, our results support the previously proposed model in which protomers play a major role in the ATPase hexamer during ATP hydrolysis. While one protomer is responsible for ATP recognition, the adjacent protomer attacks the γ-phosphate, using the R-finger to facilitate hydrolysis (Burgess et al., 2016a).

T3SS ATPases of pathogenic bacteria are considered promising targets for the development of antivirulence drugs because they are highly conserved both structurally and functionally (Marshall and Finlay, 2014; Charro and Mota, 2015). Antivirulence drugs targeting T3SS ATPase that would disarm rather than kill bacteria. Therefore, antivirulence drugs have the potential to be an important alternative or addition to classical antibiotics in future. There are a handful of ligand-bound and unliganded T3SS ATPase structures available in the protein date bank(PDB), including *Escherichia coli* O127:H6 EscN (PDB ID: 2OBM), *Salmonella typhimurium* FliH-FliI complex (PDB ID: 5B0O), and *Salmonella typhimurium* SsaN(PDB ID: 4NPH). Structural comparison of these T3SS ATPase homologs revealed that the T3SS ATPase is highly conserved (Figures S5, S2), especially the ATPase domain, which indicates that they share a similar ATP-binding site for ATP. In our study, we first captured snapshots that facilitate a complete description of nucleoside triphosphate recognition, including a bound slowly hydrolysable ATP analog ATPγS, a catalytically important Mg^2+^ ion and an ordered water molecule. Such information could facilitate the construction of a general model for designing small inhibitors targeting T3SS ATPases in various Gram-negative bacteria. In recent years, many small inhibitors targeting T3SS ATPases have been identified and developed against pathogens (Swietnicki et al., 2011; Gong et al., 2015; Grishin et al., 2018). Small-molecule compounds that bind to T3SS ATPases based on the nucleotide-bound Spa47 structure determined in the present work could obstruct the ATP-binding site and effectively inhibit catalytic activity. Our findings provide a structural basis for ATP recognition and offer valuable insight into the design of high-affinity nucleoside analog inhibitors targeting type III-associated ATPases.

## Accession codes

The atomic coordinates and structure factors have been deposited in the Protein Data Bank with the following accession codes: PDB ID: 5YBH for unliganded Spa47Δ1-83; PDB ID:5ZT1 for ATPγS bound Spa47Δ1-83; PDB ID: 5YBI for AMPPNP bound Spa47Δ1-83.

## Author contributions

SC and XG designed the study. SC and XG wrote the paper. XG and ZM purified and crystallized protein and determined structure. XG and ZM performed ATPase assay and ITC assay. BQ and XY designed construct for expression of protein. JW and MW collected date. All authors reviewed the results and approved the final version of the manuscript.

### Conflict of interest statement

The authors declare that the research was conducted in the absence of any commercial or financial relationships that could be construed as a potential conflict of interest. The reviewer YW and handling Editor declared their shared affiliation.
