# Supplementary material for: Structural Insight Into Conformational Changes Induced by ATP Binding in a Type III Secretion-Associated ATPase From Shigella flexneri
Source: Front Microbiol. 2018 Jul 2;9:1468. doi: 10.3389/fmicb.2018.01468 (PMC6036117; doi:10.3389/fmicb.2018.01468)
Supplement: Supplementary file 1 [file Data_Sheet_1.DOCX]

***Supplementary Material***

**Structural Insight into Conformational Changes Induced by ATP Binding in a Type III Secretion-Associated ATPase from *Shigella flexneri***

Xiaopan Gao^1‡^, Zhixia Mu^1‡^, Xia Yu^1^, Bo Qin^1^, Justyna Wojdyla^2^, Meitian Wang^2^ and Sheng Cui^1*^

^‡^These authors contributed equally to this work.

^*^Correspondence:

Corresponding author mailing address:

Sheng Cui, PhD,

Institute of Pathogen Biology, Chinese Academy of Medical Science

No.9 Dong Dan San Tiao, Dong Cheng Qu100730, Beijing P.R. CHINA

Email: cui.sheng@ipb.pumc.edu.cn (S.C.)

**SUPPLEMENTAL FIGURES AND TABLES**

**SI Figure 1**


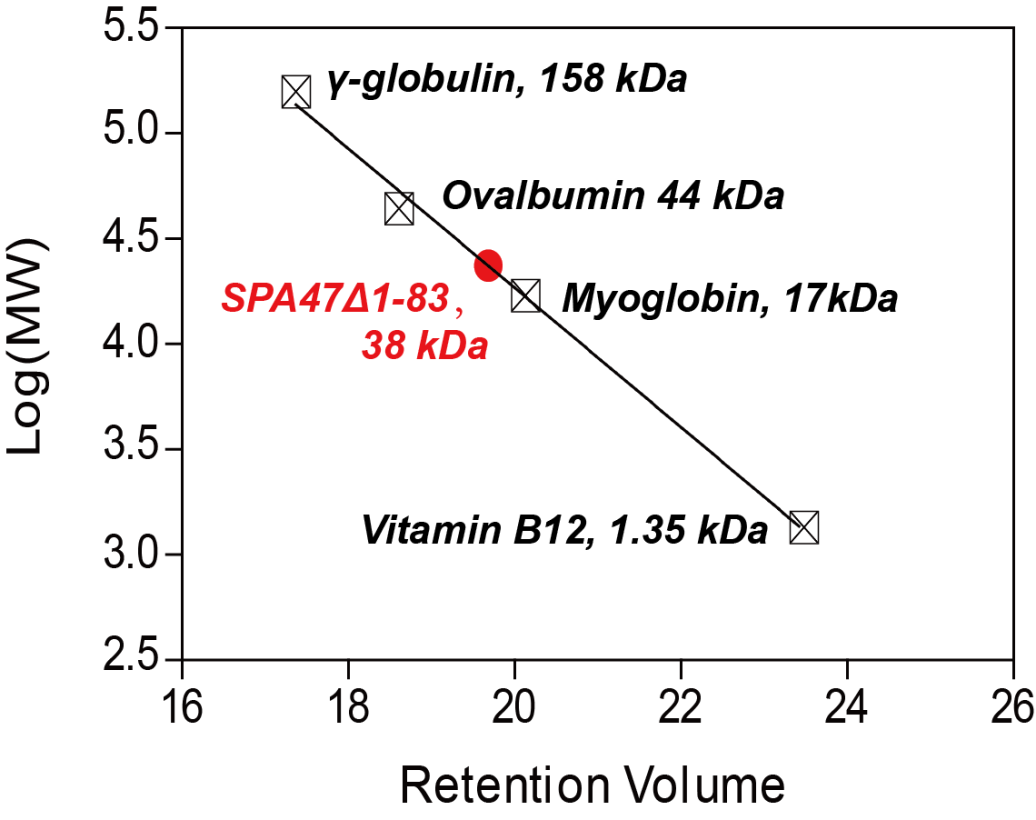


**Figure S1, Molecular weigh of Spa47Δ1-83 assessed by size exclusion chromatography**

Superdex-75 HR 10/300GL column was equilibrated with protein standards,γ-globulin 158 kDa, Ovalbumin, 44 kDa, Myoglobin17 kDa and Vitamin B12, 1.35 kDa. The molecular weight of Spa47Δ1-83 was calculated as 38kDa, indicative a monomer in solution.

**SI Figure 2**


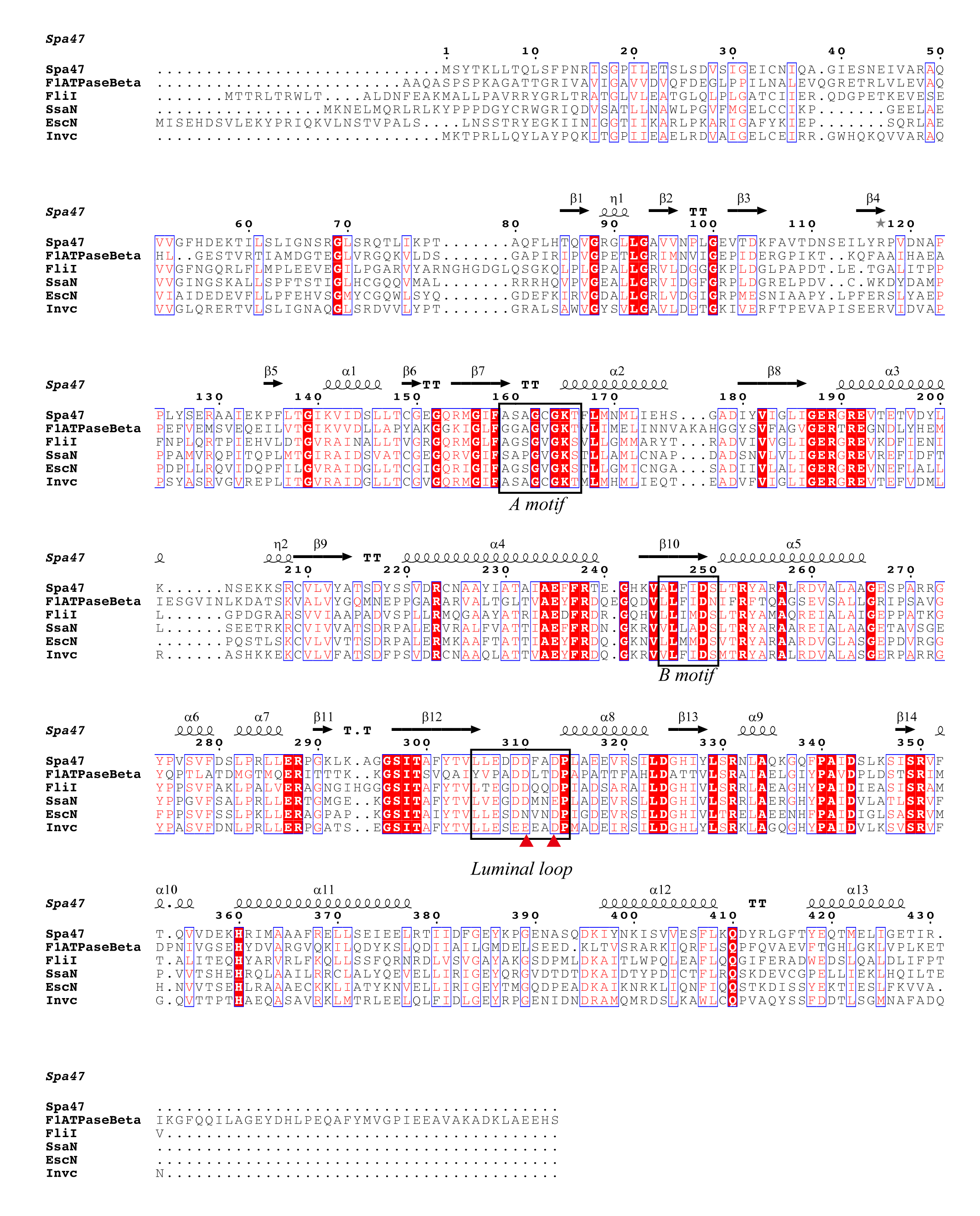


**Figure S2, Structural based multi-sequence alignment**

Structure-based multiple sequence alignment of Spa47,F_1_ATPase β subunit, FliI, SsaN, EscN, and InvC. The secondary structure of Spa47 is shown on the top, invariant residues among these proteins are highlighted with red background, conserved residues are shown in red, conserved motifs are indicated at black boxes. The alignment was carried out using the multiple sequence alignment program ClustalW and ESPript 3.0.

**SI Figure 3**


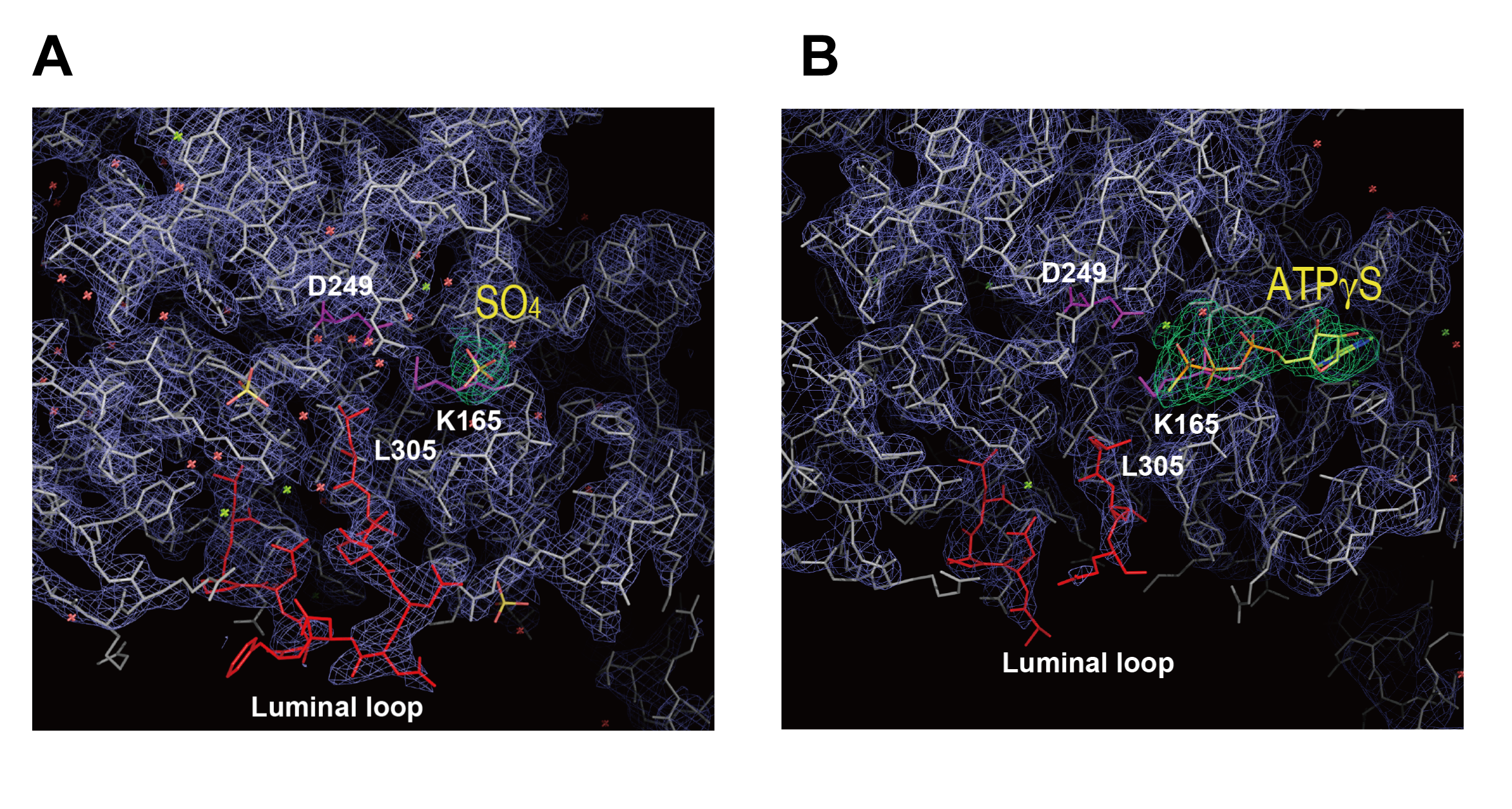


**Figure S3, Conformational changes induced by nucleotide binding.**

The magnified views of ATP binding site of Spa47 bound by ATPγS, showing the conformational changes of K165 (colored in magenta), D249 (colored in magenta), L305 and the luminal loop (residues 305-315 highlighted in red) : apo structure without ligand soaking ,(A); ATPγS,(B). The sulfate at the P-loop was from the crystallization buffer. Residues are shown in stick model with the superimposed final electron density map (2Fo-Fc, contour level 1.0). Residues recognizing nucleotides are indicated.

**SI Figure 4**


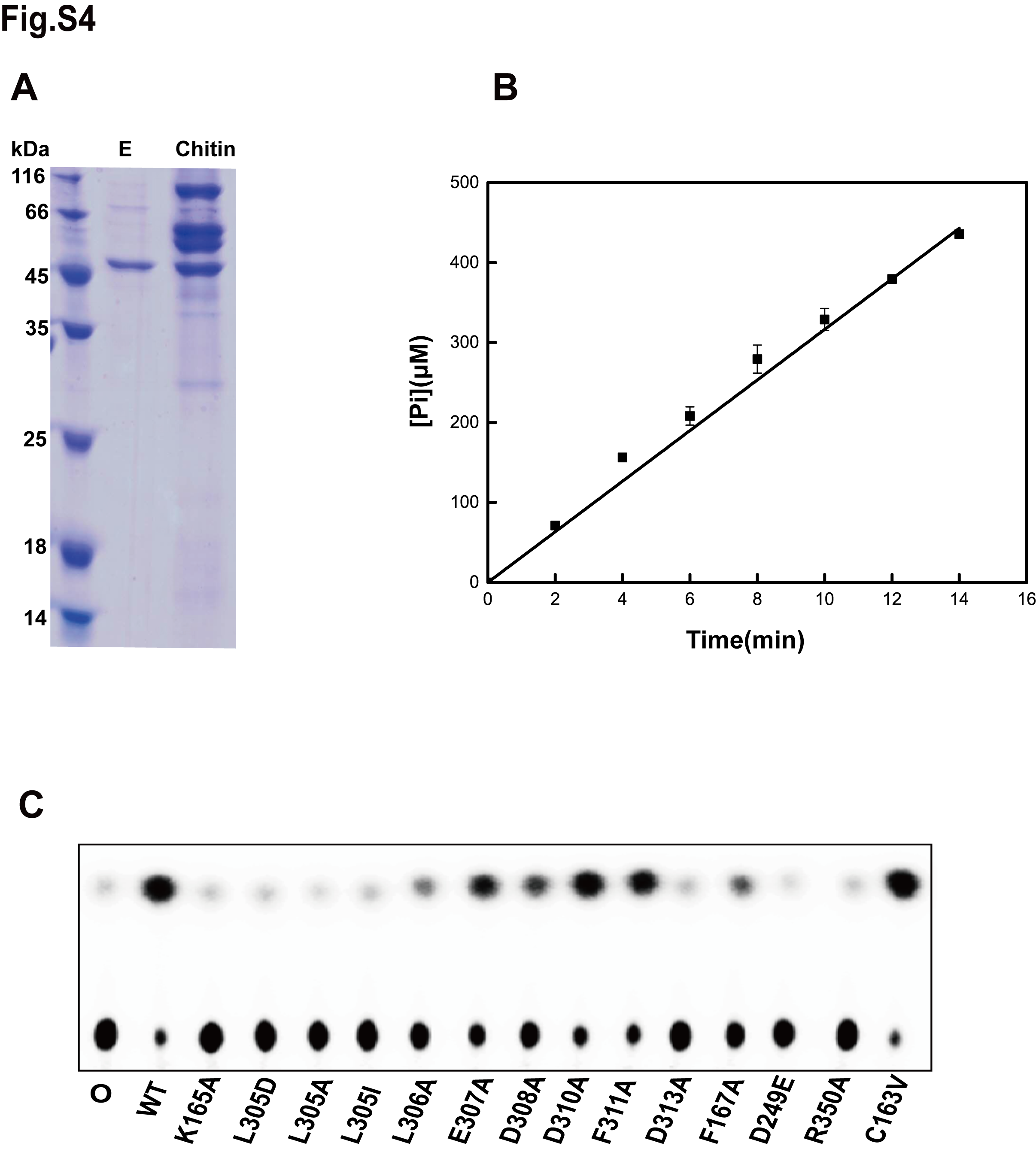


**Figure S4, Purification of full length Spa47 and ATPase activity of various mutants**

(A), SDS-PAGE of Spa47 at uncleaved CBD-Intein-Spa47 precursor (Chitin) and cleaved Spa47 stages(E).

(B), Kinetic analysis of ATP hydrolysis of wild-type Spa47.Each data point is average of three independent measurements.

(C), The raw data of thin layer chromatograph(TLC)displaying the released Pi from ATP after incubation with various mutants.

**SI Figure 5**


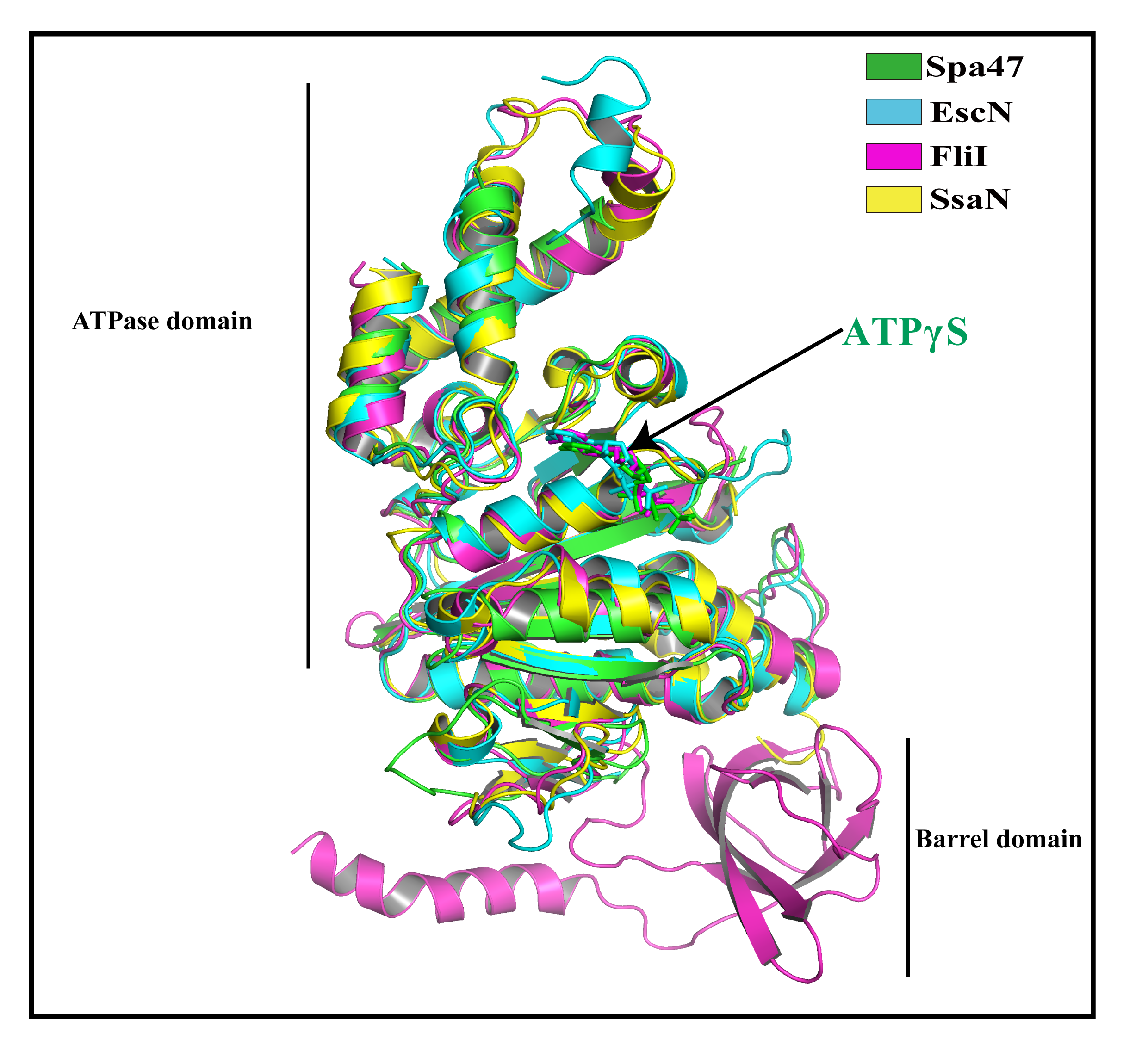


**Figure S5, Superimposition of the crystal structures of Spa47Δ1-83 and its homologues**

### Cartoon model of Spa47Δ1-83(green), EscN(cyans, PDB ID: 2OBM), FliI(magentas, PDB ID: [5B0O](http://www.rcsb.org/structure/5B0O)), SsaN(yellow, PDB ID: 4NPH)are compared with each other to illustrate the similarities between these proteins. The ATPase domain and the FliI β barrel domain are indicated; The ATPγS from Spa47Δ1-83(green) structure is also indicated.

**Tables**

**Table S1 Thermodynamic parameters of Spa47Δ1-83 binding to ATP, ATPγS, AMP-PNP and ADP by isothermal titration calorimetry.**

| ***ligand*** | ***K_a_,* 10^5^**. ***M^-1^*** | ***K_d_ , µM*** | ***ΔH, kcal/mole*** | ***ΔS, cal/mole/deg*** |
| --- | --- | --- | --- | --- |
| ATP | 0.443±0.070 | 23.070±3.44 | -4.99±0.53 | 4.50 |
| ATPγS, Mg^2+^ | 0.472±0.017 | 21.230±0.80 | -8.16±0.16 | -6.00 |
| AMP-PNP, Mg^2+^ | 0.031±0.001 | 322.482±12.60 | -7.21±0.11 | -8.20 |
| ADP, Mg^2+^ | 0.067±0.003 | 150.480±6.03 | -17.24±3.82 | -40.30 |

^a^Ka – affinity constant; standard deviation did not exceed ±10%.

^b^Kd – dissociation constant; calculated as 1/Ka.

^c^ΔH – enthalpy variation; standard deviation did not exceed ±10%.

^d^ΔS– entropy variation.
